# ^1^H-NMR Metabolomic Study of the Mushroom *Pleurotus djamor* for the Identification of Nematocidal Compounds

**DOI:** 10.3390/ph17050580

**Published:** 2024-04-30

**Authors:** Jesús Antonio Pineda-Alegría, Luis Manuel Peña-Rodríguez, Alexandre Cardoso-Taketa, José E. Sánchez, Juan Felipe de Jesús Torres-Acosta, Gloria Ivonne Hernández-Bolio, Anabel Ortiz-Caltempa, María Luisa Villarreal, Liliana Aguilar-Marcelino

**Affiliations:** 1 Centro de Investigación en Biotecnología, Universidad Autónoma del Estado de Morelos, Cuernavaca 62209, Morelos, Mexico; jesus.pinedaa@uaem.edu.mx (J.A.P.-A.); ataketa@uaem.mx (A.C.-T.); anabel@uaem.mx (A.O.-C.); 2 Unidad de Biotecnología, Centro de Investigación Científica de Yucatán, Mérida 97205, Yucatán, Mexico; lmanuel@cicy.mx; 3 Departamento de Ciencias de la Sustentabilidad, El Colegio de la Frontera Sur, Carretera Antiguo Aeropuerto Km. 2.5, Tapachula 30700, Chiapas, Mexico; esanchez@ecosur.mx; 4 Facultad de Medicina Veterinaria y Zootecnia, Universidad Autónoma de Yucatán, Km 15.5 Carretera Mérida-Xmatkuil, Mérida 97100, Yucatán, Mexico; jfj.torresacosta@gmail.com; 5 Departamento de Física Aplicada, Centro de Investigación y Estudios Avanzados (CINVESTAV), Unidad Mérida, Mérida 97205, Yucatán, Mexico; hboliog@gmail.com; 6 Centro Nacional de Investigación Disciplinaria en Salud Animal e Inocuidad, Instituto Nacional de Investigaciones Forestales Agrícolas y Pecuarias (INIFAP), Km 11 Carretera Federal Cuernavaca-Cuautla, No. 8534, Jiutepec 62550, Morelos, Mexico

**Keywords:** chemometrics, *Haemonchus contortus*, oyster mushroom, metabolomics, ^1^H-NMR, uracil

## Abstract

Due to the increasing populations of anthelmintic-resistant gastrointestinal nematodes and as a consequence of the adverse effects of synthetic drugs, this study focuses on the search for secondary metabolites with nematocidal activity from the edible mushroom *Pleurotus djamor* using The proton nuclear magnetic resonance (^1^H-NMR) metabolomics. The highest activity was shown by the ethyl acetate fractions of mycelium (EC_50_ 290.8 µg/mL) and basidiomes (EC_50_ 282.7 µg/mL). Principal component analysis (PCA) and hierarchical data analysis (HCA) of the ^1^H-NMR metabolic profiles data showed that the ethanolic extracts, the ethyl acetate, butanol, and water fractions from mycelium have different metabolic profiles than those from basidiomes, while low polarity (hexane) fractions from both stages of fungal development show similar profiles. Orthogonal partial least squares discriminant analysis (OPLS-DA) allowed the identification of signals in the ^1^H-NMR metabolic profile associated with nematocidal activity. The signals yielded via OPLS-DA and bidimensional NMR analysis allowed the identification of uracil as a component in the ethyl acetate fraction from basidiomes, with an EC_50_ of 237.7 µg/mL. The results obtained showed that chemometric analyses of the ^1^H-NMR metabolic profiles represent a viable strategy for the identification of bioactive compounds from samples with complex chemical profiles.

## 1. Introduction

In the last decade, reports of gastrointestinal nematode (GNI) populations resistant to anthelmintics in sheep have increased; as a result, research has been oriented toward the search for new control alternatives such as natural products [1,2,3]. Mushrooms of the genus *Pleurotus* are considered a traditional component in the diet of several cultures worldwide [4]. These mushrooms, besides being appreciated as food, are also used in traditional medicine due to their therapeutic properties, which include anti-inflammatory, immunomodulatory, antiviral, antimicrobial, antitumor, anticancer, antioxidant, insecticide, and antiparasitic, among others [5,6]. *Pleurotus djamor* is a pantropical mushroom that possesses the goodness of its genus, has short growing cycles, high nutritional values, and is an important source of natural products. In addition, the cultivation method has been developed using agro-industrial wastes so that basidiomes can be obtained through a sustainable methodology [7]. Therefore, the application of modern analytical approaches for the determination of the chemical composition of these medicinal mushrooms has gained interest. Metabolomics is an important tool in the study of natural products since it allows knowing the complete metabolic profile of a biological system under certain conditions, helps to identify the natural products present in the organism of interest, and can relate them with respect to their biological activity [8]. In contrast to bio-directed chemical fractionation, which has been the most widely used technique for the identification of biologically active natural products, metabolomics allows the identification and quantification of metabolites in an organism with much less experimental work. It also reduces the amount of organic solvents and biological material used for the study [9]. Metabolomics makes use of chemometric techniques, which are mathematical and statistical models that allow managing, reducing, and understanding the large amount of data provided through the chemical analysis of samples [10]. The data are mainly produced using spectroscopic and analytical techniques such as nuclear magnetic resonance (NMR), mass spectrometry (MS), gas chromatography (GC), and high-performance liquid chromatography (HPLC). At the same time, these studies provide information on the chemical composition of the analyzed samples [11]. In the present investigation, the nematocidal activity of the edible mushroom *P. djamor* was evaluated, and the ability of metabolomics to identify active compounds from complex chemical samples was determined. The proton nuclear magnetic resonance (^1^H–NMR) metabolic profiles of mycelia and basidiomes were compared using principal component analysis (PCA) and hierarchical data analysis (HCA). In addition, orthogonal partial least squares discriminant analysis (OPLS-DA) was used to correlate the metabolic profiles with the nematocidal activity of extracts and semi-purified fractions from both stages of fungal development, resulting in the identification of a natural product responsible for the activity.

## 2. Results and Discussion

### 2.1. Pleurotus djamor Extracts and Fractions with Nematocidal Activity

It can be observed that, in both stages of fungal development, the high-polarity fractions (aqueous fractions) had the highest yield. This is due to the high carbohydrate content present in *P. djamor* and the low lipid content, which would explain the lower yield of the low-polarity fractions [12,13].

Regarding the nematocidal activity, the percentages of exsheathment inhibition via mycelium and basidiomes of *P. djamor* are presented in Table 1. The **B2D** and **B2B** fractions of basidiomes were the most active, with 74 and 100% inhibition at 1200 µg/mL. The mycelial fractions with the highest activity were **M2C** and **M2B**, both showing 100% inhibition at 1200 µg/mL. With respect to the mean effective concentrations (EC_50_), they were calculated in the **B2D**, **B2B**, **M2C**, and **M2B** fractions because the rest of the extracts and fractions showed <37% inhibition (no activity). The lowest EC_50_ in basidiomes was shown by the **B2B** fraction (282.7 µg/mL), and this was selected for purification, while, in mycelium, the most active was **M2B** (290.8 µg/mL). Both fractions of medium polarity showed a lower EC_50_ than levamisole (Table 2). Previous studies showed the activity of *P. djamor* against *H. contortus* in egg-hatching inhibition and larval mortality tests [14,15]. However, this is the first report of the use of edible mushrooms to evaluate nematocidal activity using the larval exsheathment inhibition assay (LEIA). On the other hand, different extracts of various plants have been evaluated with this test, and the results obtained in the present investigation were similar [16,17,18] or better than those reported [19].

Fractions **B3B**, **B3B2**, **B3C**, **B3D**, and **B3F** obtained from vacuum liquid chromatography (VLC) showed 100% exsheathment inhibition at the concentration of 1200 µg/mL, whereas the rest presented <51% of exsheathment inhibition (Table 3). The most active fraction (**B3B**) showed an EC_50_ of 264 µg/mL. On the other hand, of the fractions **B3A**, **B3A2**, and **B3E-P**, the EC_50_ could not be calculated because they showed <13% of exsheathment inhibition, and of the remaining fractions, it was >400 µg/mL (Table 4). Compared to **B2B** and **M2B**, **B3B** was shown to be slightly more effective and was selected for further analysis. The description of the fraction codes can be found in Section 3.3 of the materials and methods and in the footnotes of the tables.

### 2.2. Metabolic Profiling and Chemometric Analysis of P. djamor

**Figure 1:**
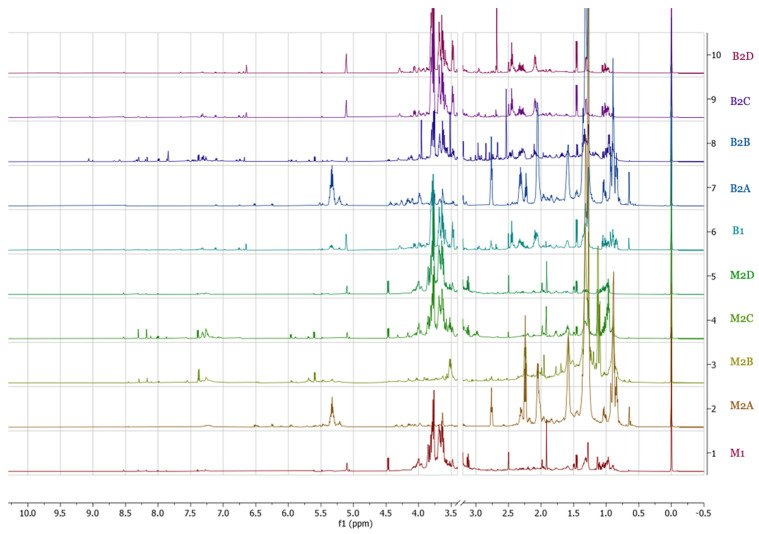
^1^H-NMR spectra of extracts and fractions of mycelium and basidiomes of *P. djamor*.

**Figure 2:**
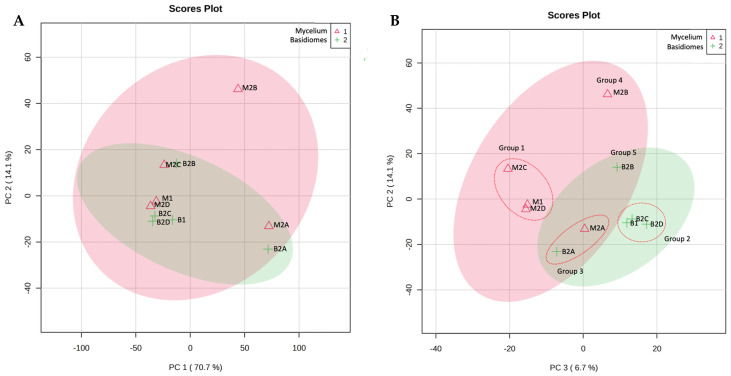
PC1–PC2 (**A**) and PC3–PC2 (**B**) score plot of ^1^H-NMR profiles of extracts and fractions of mycelium and basidiomes of *P. djamor*. Group 1: **M1**, **M2C**, and **M2D**; group 2: **B1**, **B2C**, and **B2D**; group 3: **M2A** and **B2A**; group 4: **M2B**; and group 5: **B2B**. The shaded areas indicate the 95% confidence regions based on the data points for individual groups.

The spectra obtained through ^1^H-NMR showed complex metabolic profiles in each sample, with abundant signals in the δ = 0.70–1.50 and δ = 3.50 to 4.00 regions; additionally, the ethyl acetate fractions of mycelium and basidiomes showed a higher number of signals in the δ = 5.5–9.0 regions (Figure 1). Kim et al. [20] mention that in a typical ^1^H-NMR spectrum, amino acids appear between δ = 0.5–2, primary compounds δ = 0.5–5.5, organic acids δ = 2.0–3.0, sugars δ = 3.0–5.0; while, in the aromatic region δ = 6.0–9.5, alkaloids, phenylpropanoids, flavonoids, among other secondary metabolites can be found. The binning of the ^1^H-NMR spectra into 0.04 ppm buckets resulted in a matrix with a total of 240 regions that were taken as variables and used to perform the chemometric analysis. To determine and compare the chemical profiles in the two stages of fungal development, a PCA was performed using data from crude extracts of mycelium and basidiomes (**M1** and **B1**) and their fractions (**M2A-D** and **B2A-D**), respectively. In this analysis, three principal components (PCs) were used, which together explained 91.4% of the total variance of the data. Although the PC1–PC2 plot explained 84.8%, the best separation was obtained by plotting PC3–PC2 (20.8% of the variance). Five groups could be observed in this plot: group 1: **M1**, **M2C**, and **M2D**; group 2: **B1**, **B2C**, and **B2D**; group 3: **M2A** and **B2A**; group 4: **M2B**; and group 5: **B2B**. The PCA analysis showed that crude extracts, the ethyl acetate, butanol, and water fractions from mycelium have different metabolic profiles than those from basidiomes, while low polarity (hexane) fractions from both stages of fungal development show similar profiles (Figure 2).

**Figure 3:**
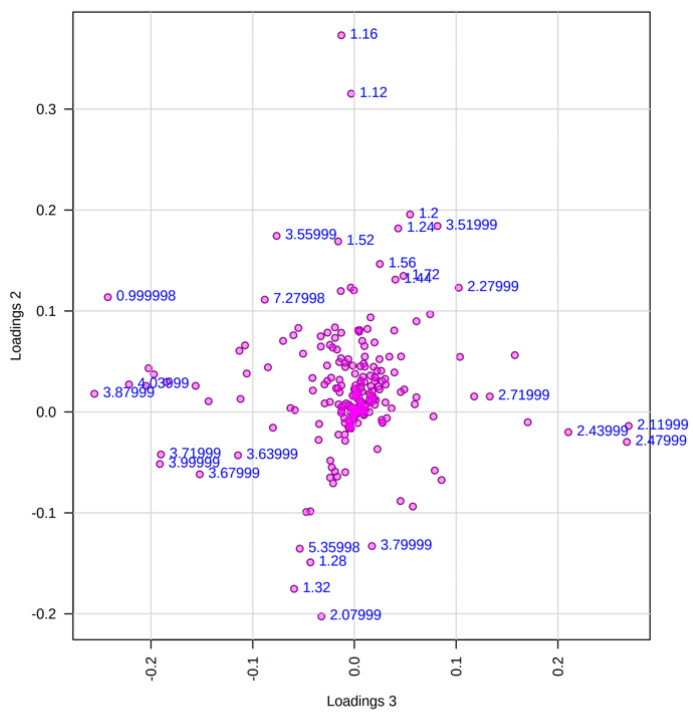
Loadings plot (PC3–PC2) of ^1^H-NMR profiles of extracts and fractions of mycelium and basidiomes of *P. djamor*. Variables with values represent the variables with the greatest influence on the separation shown in the PCA score plot.

It has been reported that during the growth and fruiting process of different *Pleurotus* species, there is a change in the composition of lignin, carbohydrates, proteins, phenolic compounds, and fats [21,22,23]. Therefore, this would explain the differences observed in the chemical profiles of the mycelial fractions with respect to those of the basidiomes. On the other hand, the fact that the hexane fractions clustered together indicates that the low-polarity compounds, such as fats and sterols, present in mycelium and basidiomes, are very similar. The variables that mainly influenced the formation of group 1 were as follows: δ = 0.99, 3.87, 4.03, 3.99, and 3.71; for group 2: δ = 2.47, 2.43, 2.11, and 2.71; for group 3: δ = 2. 07, 1.28, 1.32, and 5.35; for group 4: δ = 1.12 and 1.16; and for group 5: δ = 2.27, 3.51, 1.20, and 1.24, as can be seen in the PCA loading plot (PC3–PC2) in Figure 3.

**Figure 4:**
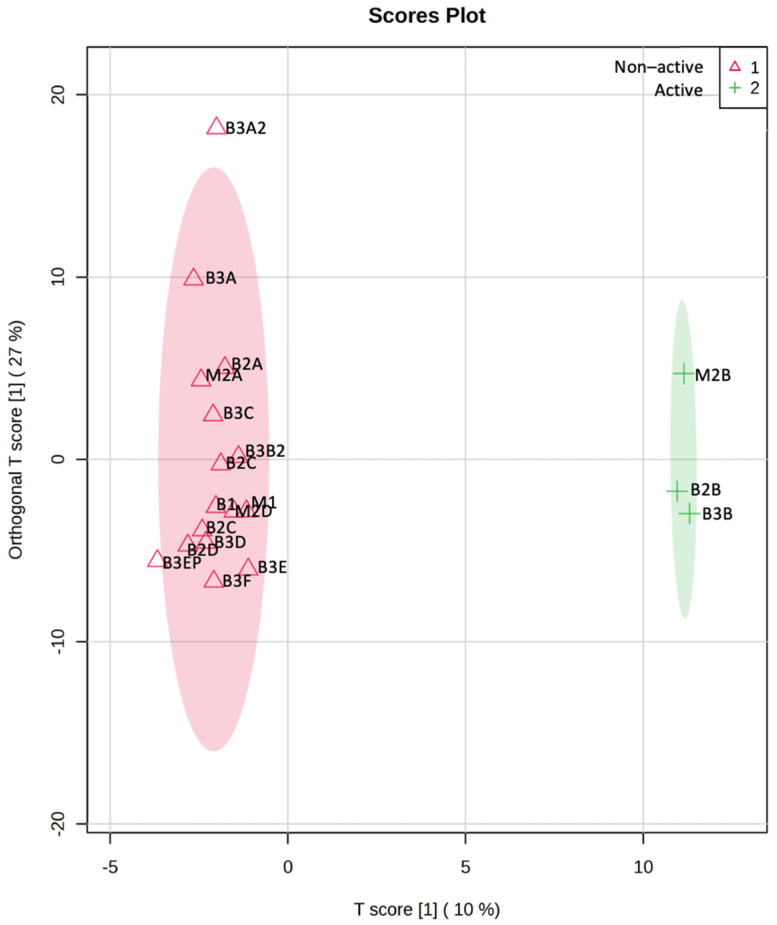
Score plot of correlation of nematocidal activity with ^1^H-NMR profiling of extracts and fractions of mycelium and basidiomes of *P. djamor*.

**Figure 5:**
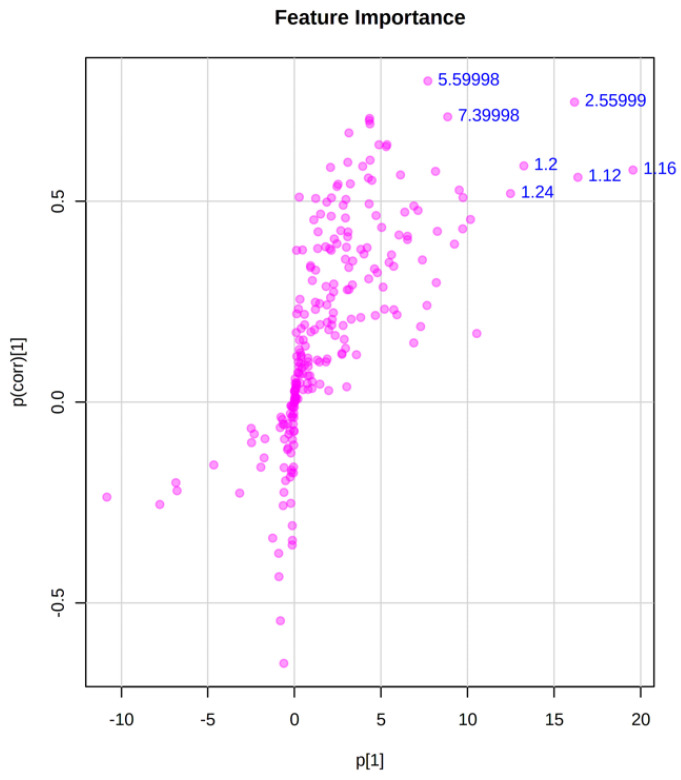
S-plot from the OPLS–DA with the variables responsible for the projection in the score plot. Variables with values represent the variables selected for further chemical identification.

**Figure 6:**
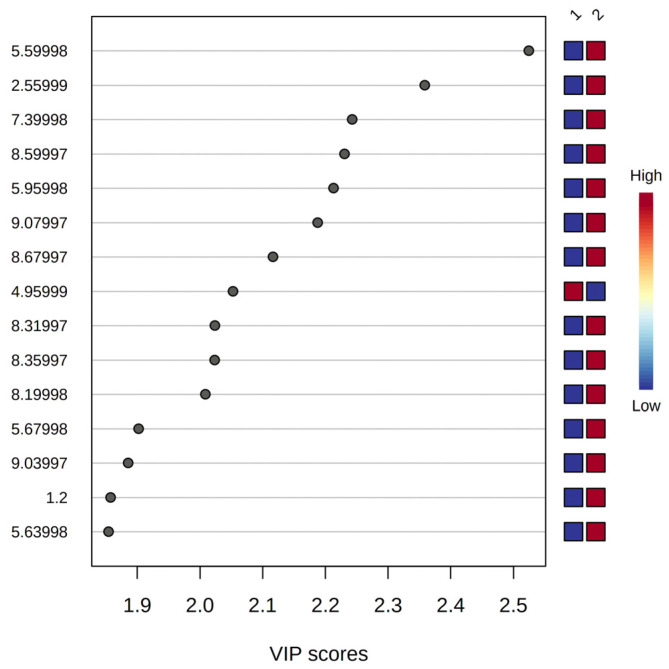
VIP plot from OPLS–DA with the variables responsible for the projection in the score plot. 1: Non active group; 2: Active group.

To identify variables associated with nematocidal activity, an OPLS-DA was performed. For this analysis, nematocidal activity data and ^1^H-NMR profiles of crude extracts, fractions, and VLC fractions were used. Those extracts/fractions having an EC_50_ < 300 µg/mL were determined as active, and the rest of them were considered non-active. The OPLS-DA score plot showed the separation of the active fractions M2B, B2B, and B3B from the rest of the non-active samples (Figure 4). The model fitting ability (R^2^) had a value of 0.78, whereas the predictive ability (Q^2^) was 0.72. The literature mentions that Q^2^ > 0.5 values are considered good, so the values obtained in this study indicate that the data fit the model and that the model is appropriate [24,25]. The identification of the variables responsible for the projection in the score plot was performed using the S-plot and the VIP plot (Variable Importance in Projection). In the S-plot, variables with a correlation >0.5 and high covariance were selected (Figure 5) [26]. On the other hand, the VIP plot showed the 15 most important variables in the projection (Figure 6) [24]. The variables selected to identify compounds with nematocidal activity according to these OPLS plots were as follows: 2.56, 5.60, 5.68, 5.96, 7.40, 8.20, 8.32, 8.36, 8.60, 8.68, 9.04, and 9.08. According to these variables, the following signals were found in the ^1^H-NMR spectra of the active fractions: **2.56:** δ = 2.55 (s); **5.60:** δ = 5.59 (d, *J* = 7.63 Hz); **5.68:** δ = 5.68 (d, *J* = 8.18 Hz); **5.96:** δ =5.95 (d, *J* = 6.41 Hz); **7.40:** δ = 7.38 (d, *J* = 7.66 Hz); **8.2:** δ = 8.2 (s), **8.32:** δ = 8.3 (s); **8.36:** δ = 8.34 (d, *J* = 7.86 Hz); **8.6:** δ = 8.6 (s); **8.68:** δ = 8.67 (s); **9.04:** δ = 9.01 (s); **9.08:** δ = 9.07 (S).

To continue with the identification of the active compounds, the signals δ = 5.59 and δ = 7.38 were selected because OPLS-DA showed that, together with δ = 2.55, they are the signals that have the greatest influence on the projection of the score plot. In addition, these signals showed the highest intensities in the ^1^H-NMR spectrum. The rest of the signals obtained through OPLS-DA (δ = 5.68, δ = 5.95, δ = 8.2, δ = 8.3, δ = 8.34, δ = 8.6, δ = 8.67, δ = 9.01, and δ = 9.07) did not show enough correlation in the bidimensional NMR analysis between ^1^H and ^13^C to allow the identification of a chemical structure. The bidimensional NMR analysis showed that the proton signal at δ = 2.55 correlates with the carbon at δ = 32.3. On the other hand, it was possible to identify a correlation between the signals at δ = 5.6 and δ = 7.38 and the carbons to which they are connected (Table 5). These ^1^H and ^13^C chemical shifts were used to search in the Biological Magnetic Resonance Data Bank (https://bmrb.io; accessed on 17 October 2023) and The Human Metabolome Database (https://hmdb.ca; accessed on 17 October 2023) for metabolites having signals with similar chemical shifts [27,28]. The search was filtered for metabolites that have been isolated from edible mushrooms, mainly from the genus *Pleurotus*. In addition, for the search and prediction of ^1^H and ^13^C chemical shifts, the freely available online programs NMRSHIFTDB2 and NMRDB2 were used [29,30].

**Figure 7:**
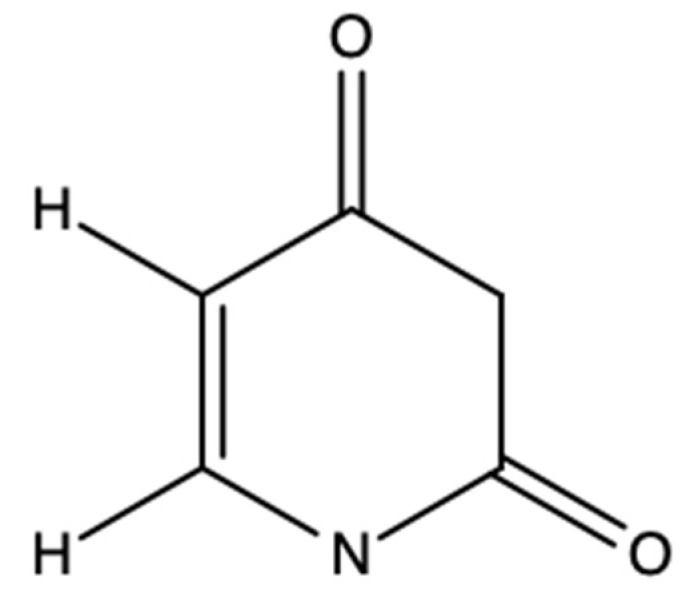
Chemical structure of Uracil.

The search results showed that the proton signals at δ = 5.6 and δ = 7.38 ppm and the ^13^C with which they are connected match the uracil chemical shifts (Table 6 and Figure 7). The differences in the chemical shifts compared to those reported in this study are minimal, and this is mainly due to the conditions under which the experiments were carried out: Still et al. [31] performed ^13^C analysis at 25.16 MHz in DMSO-d_6_; Peña-rodríguez [32] performed ^1^H analysis at 100 MHz in DMSO-d_6_; Jofre et al. [33] conducted ^1^H and ^13^C analysis at 600 MHz in D_2_O; Wishart et al. [34] performed ^1^H analysis at 500 MHz in D_2_O; and in this study, experiments were performed at 600 MHz in CD_3_OD. correlation spectroscopy (COSY), heteronuclear single quantum correlation spectroscopy (HSQC), heteronuclear connectivity to multiple bonds (HMBC), and total correlation spectroscopy (TOCSY) studies played an important role in this study since the H–H and H–C interactions (1, 2–3 bonds) obtained from these analyses match the structure of uracil. Likewise, the coupling constants (*J*) reported in this study are quite similar to those from previous experiments.

The activity of commercial uracil (CAS: 66-22-8; Sigma-Aldrich^®^, St. Louis, MO, USA) was evaluated and showed a CE_50_ of 237.7 µg/mL in *H. contortus* exsheathment inhibition. Previous studies reported the identification of uracil from a methanolic extract of *P. cornucopiae* and reported renoprotective activity under in vitro conditions [35]. It was also identified in an ethyl acetate extract obtained from *P. nebrodensis* and attributed to anticancer activity [36]. This background indicates that this metabolite may be frequently bioavailable in mushrooms of the genus *Pleurotus*. In addition to the well-known functions carried out using uracil in RNA, antihypertensive activity has been reported when combined with glycerol [37]. On the other hand, Pałasz and Cież [38] mention that different analogs, such as 5-fluorouracil or 5-chlorouracil, present mainly antiviral and antitumor activity. This is the first time that the antiparasitic activity of uracil has been reported. In this sense, further studies are needed to determine other biological capacities of uracil and its mechanisms of action.

## 3. Materials and Methods

### 3.1. Production of Mycelium of P. djamor

The edible mushroom strain *P. djamor* ECS-0123, with GenBank number GU722265, was obtained from the mycological strain bank of El Colegio de la Frontera Sur (ECOSUR) located in Tapachula, Chiapas, Mexico. The inoculum of 1 cm diameter was reseeded in Petri dishes with whole wheat flour medium (HIT). The fungal cultures were maintained in an incubator in the absence of light at a temperature of 27–30 °C. After 14 days, the mycelium was harvested by scraping the surface. Finally, the mycelium was lyophilized [39].

### 3.2. Production of Basidiomes of P. djamor

The basidiomes were produced and provided by the tropical fungi laboratory of ECOSUR. For their production, different inputs were used, such as sorghum grains (*Sorghum bicolor* L.), Pangola grass (*Digitaria decumbens*), and coffee pulp. The inoculum consisted of 2.5% *P. djamor* seed, which was homogenized and preserved in polyethylene bags with holes to allow air exchange. The bags were incubated at 85% humidity, 22 °C temperature, under natural lighting and ventilation for 40 days. At the end of this period, the fully developed basidiomes were harvested [40].

### 3.3. Preparation and Fractionation of Crude Extract of Mycelium and Basidiomes of P. djamor

The mycelium and basidiomes were placed separately in Erlenmeyer flasks, and ethanol was added. Ethanol covered all fungal material in a 3:1 ratio (solvent:fungal material), and samples were allowed to macerate for 72 h and filtered to remove solid material. Subsequently, the samples were concentrated using a rotary evaporator. The extracts were lyophilized to remove any remaining water. Once dried, 1.8 g and 1.7 g, respectively, of the ethanolic extract of mycelium (**M1**) and the ethanolic extract of basidiomes (**B1**) were stored for bioassays and chemical analysis. The **M1** extract (23.56 g) was resuspended in 250 mL of a 3:2 (*v:v*) water–methanol solution and fractionated via liquid–liquid extraction successively with hexane (3 times; 2:1, 1:1 and 1:1; *v:v* of solvent: aqueous suspension), ethyl acetate (3 times, 2:1, 1:1 and 1:1) and water-saturated butanol (once, 1:2). Each of the samples was concentrated and lyophilized to remove excess solvent and water. In this way, the hexane fraction (**M2A**; 0.74 g), ethyl acetate fraction (**M2B**; 0.40 g), butanol fraction (**M2C**; 0.31 g), and aqueous fraction (**M2D**; 22.52 g) were obtained.

On the other hand, extract **B1** (148.2 g) was resuspended in 1 L of a 3:2 (*v:v*) water–methanol solution and fractionated following the same methodology; thus, four fractions were obtained: hexane fraction (**B2A**; 28.5 g), ethyl acetate fraction (**B2B**; 7.7 g), butanol fraction (**B2C**; 35.6 g), and aqueous fraction (**B2D**; 53.8 g) [41].

The **B2B** fraction was purified via VLC following the methodology of Coll and Bowden [42]. First, 3 g of the fraction was adsorbed on silica gel 60 (0.063–0.200 mm). Filter paper (Whatman No. 1) was placed at the bottom of the column. The 4.5 cm diameter column was packed with silica gel 60 GF_254_ to a height of 5 cm. Subsequently, silica gel (1-cm high) was added, followed by absorbent cotton. The column elution system was CH_2_Cl_2_–MeOH–H_2_O (14:7:1), and the fractions collected were 100 mL. Fractionation monitoring was performed via thin-layer chromatography (TLC). At the end, an acetone wash and a methanol wash were performed. The fractions were pooled according to the TLC analysis, concentrated in a rotary evaporator, and transferred to glass vials for lyophilization drying. Finally, 9 fractions were obtained: **B3A** (344.1 mg), **B3A2** (146 mg), **B3B** (845 mg), **B3B2** (20 mg), **B3C** (342 mg), **B3D** (230 mg), **B3E** (757 mg), **B3E-P** (80 mg), and **B3F** (77 mg).

### 3.4. Evaluation of Nematocidal Activity

The infecting larvae (L_3_) of *H. contortus* used for this bioassay were from the isolate “paraíso”; this isolate was reported to be resistant to benzimidazole [43]. For the production of L_3_ larvae, a donor sheep artificially infected with 4000 L_3_ orally was used. After 21 days, feces were collected from the rectum of the sheep, and stool culture was performed. After seven days, larvae were recovered using the Baermann funnel technique; excess feces were removed by washing with distilled water and stored at 4 °C until use [17]. Evaluation of nematocidal activity was carried out using the LEIA. For the assay, 1000 L3 larvae of *H. contortus* were placed in 15 mL tubes and incubated at different concentrations of each extract/fraction (100, 200, 400, 400, 600, and 1200 µg/mL) for 3 h at 23 °C in 2 mL. In addition, PBS (pH: 7.2) or Tween (1%) without extract was included as a negative control. Commercial levamisole (Vermidazole-15, Prossiter S.A de C.V, Atizapán de Zaragoza, México) was used as a positive control. Subsequently, the larvae were subjected to three washes with PBS via centrifugation (3500 rpm, 3 min). For this, after each centrifugation, 1 mL of the treatment was removed, and 1 mL of PBS was added. Then, 200 µL aliquots were made in Eppendorf tubes. Four replicates of each treatment and negative control were carried out. Subsequently, the larvae were artificially induced with a solution of sodium hypochlorite (4–6%) and sodium hydroxide (0.02–0.1%) diluted with PBS at 1/200, 1/240, and 1/300. For bioassay readings, 50 µL were added on slides and coverslips. Inhibition of larval exsheathment was determined through microscopic observation (10×) at 0, 20, 40, and 60 min. The unsheathing process was stopped each time by flaming the slides containing the larvae [43]. Finally, the number of larvae with and without sheath was recorded, and the percentage of exsheathment inhibition was calculated using the following formula:

(1) $$\mathrm{Exsheathment}\;\mathrm{inhibition}\;\left(\%\right)=\frac{\mathrm{number}\;\mathrm{of}\;\mathrm{larvae}\;\mathrm{without}\;\mathrm{sheath}}{\mathrm{number}\;\mathrm{of}\;\mathrm{larvae}\;\mathrm{with}\;\mathrm{sheath}\;+\;\mathrm{number}\;\mathrm{of}\;\mathrm{larvae}\;\mathrm{without}\;\mathrm{sheath}}\times 100.$$

The analysis of the results was carried out via a one-way analysis of variance, followed by Tukey’s mean comparison test (*p* < 0.05). Finally, for each of the extracts/fractions of mycelium and basidiomes of *P. djamor*, the mean effective concentration (EC_50_) to inhibit larval exsheathment was determined. The analyses were performed with R software (V.3.6.3).

### 3.5. ^1^H-NMR Metabolic Profiling and Multivariate Analysis

First, 10 mg of each *P. djamor* mycelial and basidiome extracts and fractions were diluted in 600 µL of deuterated methanol (CD_3_OD) with 0.05% sodium 3-trimethylsilyl propionate (TSP) and placed in 5 mm NMR tubes. Metabolomic profiles were determined through ^1^H-NMR analysis at 600 MHz. Spectroscopic data were subjected to preprocessing with MestReNova 14.2 software (Mestrelab Research SL, Santiago de Compostela, Spain). The phase and baseline were manually adjusted; in addition, the scale of the chemical shift with respect to the TSP signal was calibrated to 0 ppm, and the spectra were stacked. Then, a cutoff was performed in the region of δ = 3.2–3.4, corresponding to the CD_3_OD signal, and the spectral intensities were scaled with respect to the TSP. Finally, the spectra were split into 0.04 ppm buckets from the δ = 0.2–10 region and exported as a comma-separated value (CSV) file [44]. The chemometric analysis of the data was performed using MetaboAnalyst V.4.0 (https://www.metaboanalyst.ca, accessed on 24 March 2023), where HCA, PCA [45,46], and OPLS-DA were carried out, allowing the correlation of the signals of the ^1^H-NMR profiles with the biological activity data [24]. For the identification of the active metabolites, the ^1^H-NMR signals selected via OPLS-DA were used, and ^13^C analysis, COSY, HSQC, HMBC and TOCSY were performed on the sample with the highest nematocidal activity [11].

## 4. Conclusions

The ethyl acetate fractions showed the highest nematocidal activity in both stages of fungal development.

The chemometric analysis of the ^1^H-NMR metabolic profile data, combined with the bioactivity data, allowed for the identification of signals in the spectra associated with nematocidal activity. When combined with the bidimensional NMR analysis, uracil was determined as the active compound.

^1^H-NMR metabolomics proved to be an important tool for determining the chemical composition and identifying active nematocidal compounds from chemically complex samples without requiring previous knowledge of the chemical content.

## Figures and Tables

**Table 1:** Exsheathment inhibition of *Haemonchus contortus* L_3_ larvae via the effect of basidiomes and mycelium of *Pleurotus djamor* and levamisole.

Concentration (µg/mL)	Mycelium					Basidiomes					Anthelmintic
	M1	M2A	M2B	M2C	M2D	B1	B2A	B2B	B2C	B2D	Levamisole
PBS	3.1 ^b^		–	3.3 ^c^	1.7 ^b^	2.1 ^b^	–	–	0 ^a^	0 ^c^	2.6 ^c^
Tween 1%	–	0 ^b^	1.6 ^d^	–	–	–	0 ^b^	2.1 ^d^	–	–	–
100	12.5 ^ab^	2.4 ^b^	17.4 ^cd^	18.7 ^b^	18.4 ^ab^	12.3 ^ab^	3.0 ^b^	33.8 ^c^	0 ^a^	16.2 ^bc^	2.1 ^c^
200	20.6 ^ab^	5.9 ^ab^	23.9 ^c^	23.8 ^b^	21.8 ^ab^	15.2 ^ab^	8.3 ^ab^	36.0 ^c^	5.7 ^a^	16.9 ^bc^	8.5 ^c^
400	27.7 ^a^	10.4 ^ab^	52.3 ^bc^	31.3 ^b^	22.5 ^ab^	18.9 ^a^	11.0 ^ab^	44.4 ^c^	12.2 ^a^	27.3 ^bc^	67.5 ^b^
600	27.1 ^a^	11.9 ^ab^	77.3 ^b^	31.2 ^b^	24.6 ^a^	26.0 ^a^	11.5 ^ab^	69.1 ^b^	15.8 ^a^	36.7 ^ab^	94.0 ^a^
1200	36.9 ^a^	26.8 ^a^	100 ^a^	100 ^a^	29.6 ^a^	28.4 ^a^	29.3 ^a^	100 ^a^	17.2 ^a^	74.1 ^a^	100 ^a^
MSE	3.1	2.8	6.4	5.5	2.7	2.3	6.1	5.7	2.7	4.8	7.5

**M1**: Ethanolic extract of mycelium; **M2A**: Aqueous fraction of mycelium; **M2B**: Butanol fraction of mycelium; **M3C**: Ethyl acetate fraction of mycelium; **M2D**: Hexane fraction of mycelium; **B1**: Ethanolic extract of basidiomes; **B2A**: Aqueous fraction of basidiomes; **B2B**: Butanol fraction of basidiomes; **B2C**: Ethyl acetate fraction of basidiomes; **B2D**: Hexane fraction of basidiomes. Percentages were determined using data from minute 60. MSE: Mean standard error. Different letters in the same column indicate statistically significant differences (*p* < 0.05).

**Table 2:** Mean effective concentrations (EC_50_) with 95% confidence intervals (95%CI) of different extracts and fractions of basidiomes and mycelium of *Pleurotus djamor* and levamisole, necessary to inhibit *Haemonchus contortus* larval exsheathment.

Extract/Fraction	EC_50_ (IC95%) µg/mL
Mycelium	
**M1**	No activity
**M2A**	No activity
**M2B**	290.8 (251.0–330.6)
**M3C**	537.5 (434.4–640.5)
**M3D**	No activity
Basidiomes	
**B1**	No activity
**B2A**	No activity
**B2B**	282.7 (230.8–334.6)
**B2C**	No activity
**B2D**	782.2 (494.7–1069.7)
Anthelmintic	
levamisole	315.4 (287.1–343.7)

**M1**: Ethanolic extract of mycelium; **M2A**: Aqueous fraction of mycelium; **M2B**: Butanol fraction of mycelium; **M3C**: Ethyl acetate fraction of mycelium; **M2D**: Hexane fraction of mycelium; **B1**: Ethanolic extract of basidiomes; **B2A**: Aqueous fraction of basidiomes; **B2B**: Butanol fraction of basidiomes; **B2C**: Ethyl acetate fraction of basidiomes; **B2D**: Hexane fraction of basidiomes. EC_50_ was determined using data from minute 60.

**Table 3:** Exsheathment inhibition of *Haemonchus contortus* L_3_ larvae (Mean ± MSE) via the effect of fractions obtained from **B2B** purification through VLC.

Concentration (µg/mL)	B3A	B3A2	B3B	B3B2	B3C	B3D	B3E	B3F	B3E–P	Levamisole
PBS	0 ^b^	–	0 ^d^	–	0 ^d^	0 ^c^	0 ^b^	0 ^d^	0 ^a^	2.6 ^c^
Tween 1%	–	0 ^a^	–	0 ^c^	–	–	–	–	–	–
100	0 ^b^	0 ^a^	6.6 ^cd^	0 ^c^	0 ^d^	1.0 ^c^	0.8 ^b^	0 ^d^	0 ^a^	2.1 ^c^
200	0 ^b^	0 ^a^	13.0 ^c^	0 ^c^	1.6 ^d^	2.1 ^c^	0.9 ^b^	0 ^d^	0 ^a^	8.5 ^c^
400	0 ^b^	0 ^a^	89.1 ^b^	28.7 ^b^	17.1 ^c^	13.5 ^c^	1.3 ^b^	8.1 ^c^	0 ^a^	67.5 ^b^
600	3.8 ^ab^	0 ^a^	100 ^a^	98.4 ^a^	74.6 ^b^	45.9 ^b^	7.1 ^b^	50.8 ^b^	0 ^a^	94.0 ^a^
1200	13 ^a^	0 ^a^	100 ^a^	100 ^a^	100 ^a^	100 ^a^	52.4 ^a^	100 ^a^	0 ^a^	100 ^a^
MSE	1.9	0	7.9	8.4	7.1	6.8	3.6	7.0	0	7.5

Percentages were determined using data from minute 60. MSE: Mean standard error. Different letters in the same column indicate statistically significant differences (*p* < 0.05).

**Table 4:** Mean effective concentrations (EC_50_) with 95% confidence intervals (95%CI) of the fractions obtained from the VLC to inhibit *Haemonchus contortus* larvae exsheathment.

Fraction	CE_50_ (IC95%) µg/mL
**B3A**	No activity
**B3A2**	No activity
**B3B**	264.0 (239.8–288.2)
**B3B2**	417.3 (401.6–433.0)
**B3C**	500.5 (454.3–546.7)
**B3D**	591.7 (549.1–634.2)
**B3E**	1171.0 (1048.1–1294.0)
**B3F**	574.2 (534.3–614.1)
**B3E-P**	No activity
levamisole	315.4 (287.1–343.7)

EC_50_ was determined using data from minute 60.

**Table 5:** Selected ^1^H signals from OPLS-DA and their correlations showed in COSY, TOCSY, HSQC, and HMBC analysis.

^1^H (ppm)	COSY (ppm)	TOCSY (ppm)	HSQC (ppm)	HMBC (ppm)
2.55 (s)	ND	ND	32.3	ND
5.60 (d, *J* = 7.63 Hz)	7.38	7.38	103.7	145.5, 169.3
7.38 (d, *J* = 7.66 Hz)	5.60	5.60	145.5	103.7, 155.4, 169.3

ND: Not detected.

**Table 6:** ^1^H and ^13^C chemical shifts of uracil.

Source	H–C5	H–C6	C2	C4	C5	C6	Reference
Other studies	NR	NR	152.7	165.2	100.9	143.0	[31]
	5.45 (d, *J* = 8 Hz)	7.4 (d, *J* = 8 Hz)	NR	NR	NR	NR	[32]
BMRB (bmse000940)	5.79	7.52	155.93	170.29	103.79	146.26	[33]
HMDB (HMDB0000300)	5.79 (d, *J* = 7.68 Hz)	7.52 (d, *J* = 7.68 Hz)	152.16 ^b^	164.8 ^b^	100.63 ^b^	142.63 ^b^	[34]
This study	5.6 (d, *J* = 7.63 Hz)	7.38 (d, *J* = 7.66 Hz)	155.49	169.32	103.7	145.56	

^b^ Data from a predictive model ^1^H-NMR (600 MHz, D_2_O). NR: Not reported.

## Data Availability

The datasets used and/or analyzed during the current study are available from the corresponding author on reasonable request.
